# Development and Notch Signaling Requirements of the Zebrafish Choroid Plexus

**DOI:** 10.1371/journal.pone.0003114

**Published:** 2008-09-03

**Authors:** Brent R. Bill, Darius Balciunas, Joshua A. McCarra, Eric D. Young, Toua Xiong, Ashley M. Spahn, Marta Garcia-Lecea, Vladimir Korzh, Stephen C. Ekker, Lisa A. Schimmenti

**Affiliations:** 1 Department of Genetics, Cell and Development, University of Minnesota, Minneapolis, Minnesota, United States of America; 2 Developmental Biology Center, University of Minnesota, Minneapolis, Minnesota, United States of America; 3 Institute of Human Genetics, University of Minnesota, Minneapolis, Minnesota, United States of America; 4 Department of Biology, Temple University, Philadelphia, Pennsylvania, United States of America; 5 Laboratory of Fish Developmental Biology, Cancer and Developmental Cell Biology Division, Institute of Molecular and Cell Biology, Agency for Science, Technology and Research, Singapore, Singapore; 6 Department of Biochemistry and Molecular Biology, Mayo Clinic, Rochester, Minnesota, United States of America; 7 Department of Pediatrics, Genetics and Metabolism, University of Minnesota, Minneapolis, Minnesota, United States of America; 8 Department of Ophthalmology, University of Minnesota, Minneapolis, Minnesota, United States of America; Centre de Regulacio Genomica, Spain

## Abstract

**Background:**

The choroid plexus (CP) is an epithelial and vascular structure in the ventricular system of the brain that is a critical part of the blood-brain barrier. The CP has two primary functions, 1) to produce and regulate components of the cerebral spinal fluid, and 2) to inhibit entry into the brain of exogenous substances. Despite its importance in neurobiology, little is known about how this structure forms.

**Methodology and Principal Findings:**

Here we show that the transposon-mediated enhancer trap zebrafish line Et^Mn16^ expresses green fluorescent protein within a population of cells that migrate toward the midline and coalesce to form the definitive CP. We further demonstrate the development of the integral vascular network of the definitive CP. Utilizing pharmacologic pan-notch inhibition and specific morpholino-mediated knockdown, we demonstrate a requirement for Notch signaling in choroid plexus development. We identify three Notch signaling pathway members as mediating this effect, *notch1b*, *deltaA*, and *deltaD*.

**Conclusions and Significance:**

This work is the first to identify the zebrafish choroid plexus and to characterize its epithelial and vasculature integration. This study, in the context of other comparative anatomical studies, strongly indicates a conserved mechanism for development of the CP. Finally, we characterize a requirement for Notch signaling in the developing CP. This establishes the zebrafish CP as an important new system for the determination of key signaling pathways in the formation of this essential component of the vertebrate brain.

## Introduction

The choroid plexus (CP) is a set of vital structures in the brain central to the formation, regulation and protection of the cerebral spinal fluid (CSF). Development and physiology of the CP has implications for many central nervous system pathologies and for the targeting of pharmaceutical treatment to the central nervous system [1], [2]. In humans and other mammals, the CP consists of four independent structures, one in each of the four ventricles of the brain [1]. On the cellular level, the structure can be divided into three separate compartments, a vascular core, a stroma, and a layer of polarized epithelia [1]. The polarized epithelial cells surround the stroma and vascular core and exist as a monolayer, with their basal surface facing the stroma, and their apical surface extending microvilli and cilia into the CSF-filled ventricles [3].

Classic studies in human embryology place the onset of CP development at 6 weeks gestation and continuing past birth [1]. Comparative studies in mice place development of the CP beginning at embryonic day 11 [4]. In humans, the first of the choroid plexuses to develop is the fourth ventricle CP (4vCP), or its orthologue the myelencephalic choroid plexus (mCP), in other organisms including telosts [1], [5]. However, despite its importance in neurobiology, little is known about how this important structure forms.

We use the transgenic line Et^Mn16^, which express GFP in a subset of definitive mCP epithelial cells, to characterize the development of the zebrafish mCP *in vivo*. The subset of the mCP primordial epithelia are initially found in a diffuse pattern along the dorsal roof of the fourth ventricle and coalesce at the midline to form the definitive mCP. Upon coalescence, the dorsal longitudinal vein (DLV) provides the small vessel bed that invades the epithelium of the mCP. Time-dependent inhibition of Notch signaling defines a requirement for this pathway in CP development. Morpholino-based ligand/receptor inhibition studies map this critical signaling event to the *notch1b* receptor and the two candidate ligands, *dla* and *dld*. Together, this study provides the first description of CP development *in vivo* and demonstrates a role for Notch signaling in mCP development.

## Results

### Identification of the zebrafish CP

**Figure 1:**
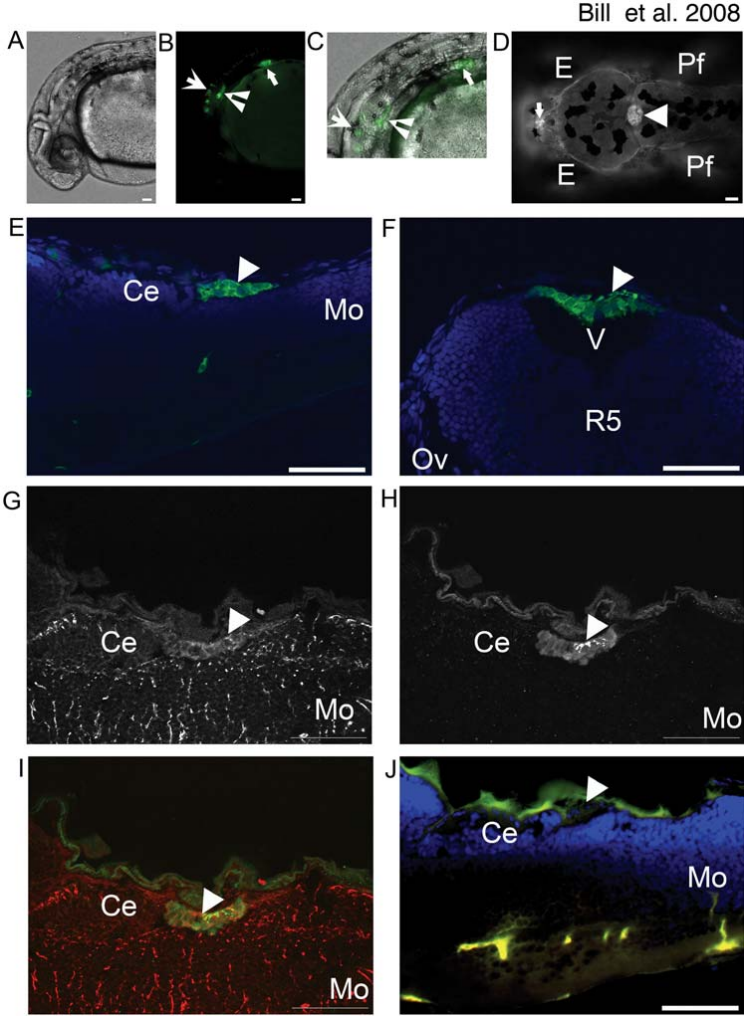
Et^Mn16^ co-localizes with Gfap in mCP epithelia. GFP is expressed in multiple anatomical locations in Et^Mn16^ larvae (A–D). At 30 hpf (A–C)(lateral orientation), GFP is expressed in cells of the otic vesicle (inverted arrowhead), the pectoral fin (closed arrow), ventral hindbrain and spinal cord (open arrow) brightfield image (A), fluorescent image (B), and merged image (C). In addition to cells of the ventral hindbrain, at 4 dpf (D)(dorsal orientation), two additional structures express GFP, the diencephalic CP (dCP) (closed arrow), and the myelencephalic CP (mCP) (closed arrowhead). The mCP lies posterior to the cerebellum (Ce) [(5 dpf, 6 µm longitudinal cryosection) (E)], within the ventricle (v) dorsal to the fifth rhombomere (r5)[5 dpf, 6 µm transverse cryosection (F)]. Cells of the mCP express Gfap (G) and colocalize with GFP expressing cells (H), merged image (I) and are not seen in the negative control lacking antibodies for Gfap and GFP (J). Abbreviations: eye (E), pectoral fin (Pf), cerebellum (Ce), and medulla oblongata (Mo). All images except F are oriented anterior to the left; F is oriented dorsal to the top. Scale bar is 50 µm.

Et^Mn16^ is a GFP expressing transposon-mediated enhancer trap line identified as part of an ongoing genomic annotation project [6]. At 30 hours post fertilization (hpf), cells of the fin bud, a symmetric set of cells that extend processes medially in the ventral hindbrain and spinal column, and the otic vesicles express GFP (Fig. 1A–C). At 4 days post fertilization (dpf), we observe two additional structures expressing GFP. The first structure appears at the dorsal-most point of the fourth ventricle on the midline (Fig. 1D, arrowhead). The second structure is situated anterior to the eyes at the dorsal-most point of the third ventricle on the midline (Fig. 1D, arrow). Based on comparative anatomical studies in multiple organisms including telosts [1], we attribute the anterior GFP-expressing cells as those of the diencephalic choroid plexus (dCP), and the posterior GFP-expressing cells as those of the myelencephalic choroid plexus (mCP) [Fig. 1E (lateral section), Fig. 1F (transverse section)].

Glial fibrillary acidic protein (Gfap) is an early transient marker of the CP epithelia [7]. We used immunohistochemistry on 6 µm longitudinal cryosections of 5 dpf mCP with a monoclonal antibody (zrf-1) directed against zebrafish Gfap [8]. We observe Gfap expression in the epithelial cells of the mCP (Fig. 1G) as defined by colocalization of Gfap (red) and GFP (Fig. 1H, merged 1J). This colocalization of Gfap and GFP-expressing cells in Et^Mn16^ larvae in conjunction with anatomical analyses indicates Et^Mn16^ labels the forming zebrafish mCP.

### Development of the mCP as visualized in the Et^Mn16^ Larvae

**Figure 2:**
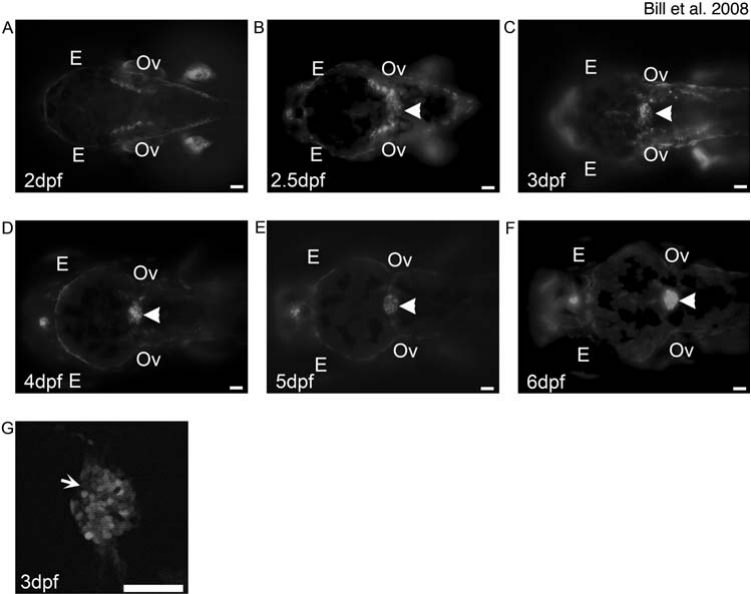
Development of the mCP as defined by Et^Mn16^ larvae. Fluorescent images of dorsal-oriented live zebrafish taken at 2 dpf (A), 2.5 dpf (B), 3 dpf (C), 4 dpf (D), 5 dpf (E), and 6 dpf (F); arrowhead indicates mCP. GFP-expressing cells first appear diffusely across the roof plate of the fourth ventricle (B), as defined by the level of the otic vesicles (Ov). Cells migrate toward the midline and finish coalescence by 4 dpf. The expression in this structure remains static through 6 dpf. Not all cells of the mCP express GFP (G, arrow). In all images, anterior is to the left, and scale bar is 50 µm. Abbreviations: eye (E), otic vesicle (Ov).

CP formation was imaged *in vivo* using Et^Mn16^ zebrafish larvae. GFP-expressing presumptive mCP are first observed in the roof plate of the fourth ventricle at 2.5 dpf (Fig. 2B). At 3 and 4 dpf, the cells progressively coalesce (Fig. 2C,D) toward the midline forming a tight rounded structure by 5 dpf (Fig. 2E). A subset of the cells within the mCP do not express GFP at this time (Fig. 2G, arrowhead). The structure remains visibly unchanged at 6 dpf, indicating the labeling of the definitive zebrafish CP (Fig. 2F).

### The vasculature of the mCP is supplied by the DLV

**Figure 3:**
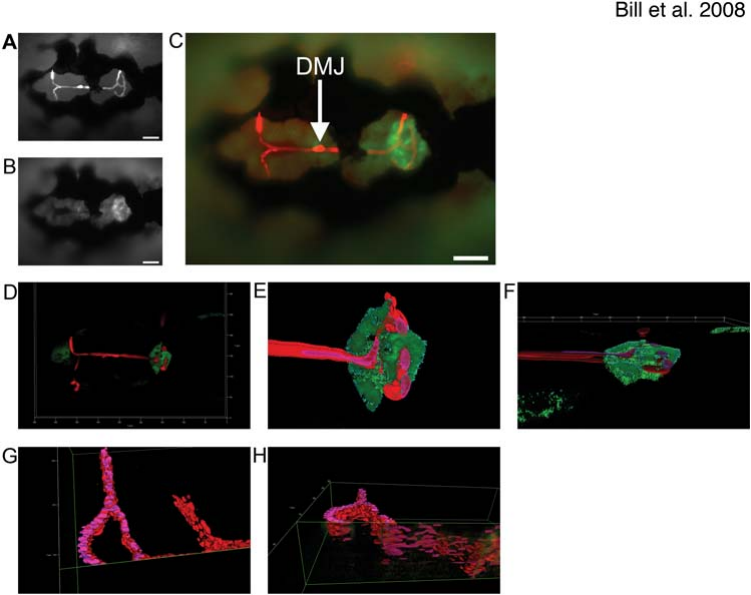
The Dorsal Longitudinal Vein supplies the mCP with its vascular network. The Dorsal Longitudinal Vein (DLV) closely associates with the mCP epithelia as defined by doubly transgenic Et^Mn16^/Tg(*gata1*:dsRed) dorsally-mounted 5 dpf live zebrafish, epithelia express GFP (A) and red blood cells express DsRed (B) merged image (C) (scale bars are 50 µm). The vessels, as defined by blood flow, directly contact the dorsal surface of the mCP. Low magnification of a 5 dpf dorsally mounted live Et^Mn16^/Tg(*gata1*:dsRed), high magnification three-dimensional renders of DLV/PCeV junctions and TCB of zebrafish shown in D (E,F). Panel F is rotated 80° into the plane to show association (dorsal facing the top of the image). The DLV bends dorsally slightly before the bifurcation, traverses the mCP epithelial domain, and then turns ventrally as it transitions to the PCeV and PCeV′. Panels G and I are three-dimensional renders of the DLV, PCeV and TCB [(G,I – 85° rotation into the plane)(oriented with anterior to the right)]. The mCP has been digitally removed, and the DLV has been bisected utilizing the clipping tools of Image4D in order to define the vascular structure. Scale is as indicated on each render.

The CP is tightly associated with vasculature in mammalian systems [9] as part of the blood brain barrier. We visualized functional blood vessel integration in the CP using Et^Mn16^/Tg(*gata1*:dsRed) double transgenic fish, with the latter transgene expressing DsRed in the circulating red blood cells [10]. Blood vessel identities were established utilizing the zebrafish vasculature map [11]. At 5 dpf, the mCP is at the bifurcation of the Dorsal Longitudinal Vein (DLV) as this vessel progresses into the Posterior Cerebral Vein (PCeV and PCeV′, prime indicates opposing bilateral side) (Fig. 3A–C). The bifurcation occurs shortly after the entry into the mCP domain. Three-dimensional renderings of the blood flowing through the DLV and mCP demonstrate that the vein is tightly associated with the CP epithelia (Fig. 3D–F). In addition, the DLV makes a slight dorsal bend just before the bifurcation, locating this vessel on the dorsal side of the mCP opposite the ventricle (Fig. 3G–H). The DLV then flattens as it joins the PCeV and PCeV′, transiting the mCP. After the PCeV and PCeV′ exit the mCP, these vessels again turn ventrally.

**Figure 4:**
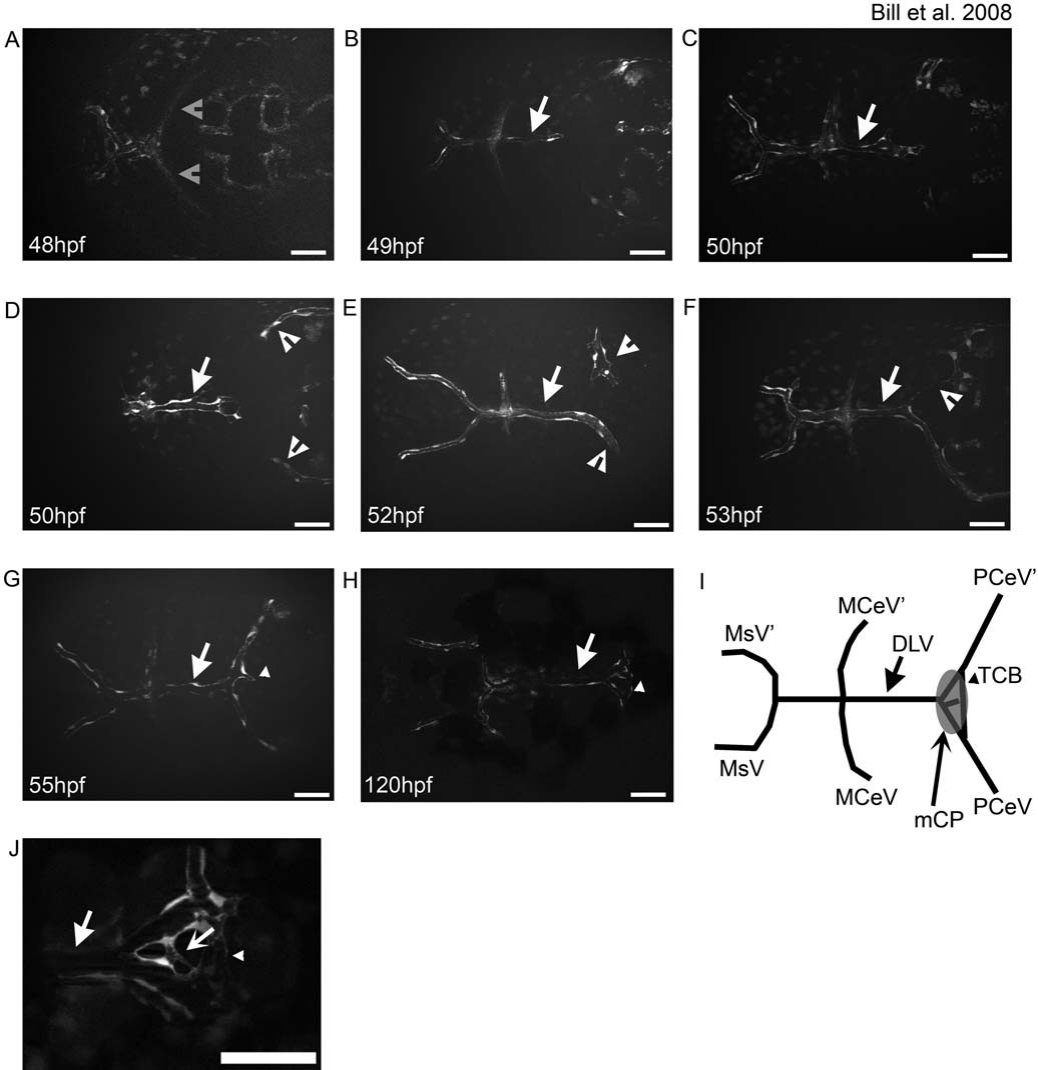
The DLV develops via angiogenic sprouting and supplies the mCP. The development of the integral vasculature in the mCP was examined in living zebrafish larvae. The DLV (arrow in all panels) sprouts from the MCeV/MCeV′ (gray open arrowheads) via angiogenic sprouting between 40 hpf (A) and 48 hpf (B), and develops via growth cone-like filopodial extensions (C). The PCeV and PCeV′ (white open arrowheads) grow dorsomedially to meet the DLV in the roof of the fourth ventricle (D). Fusion occurs between the DLV (arrow) and PCeV or PCeV′ (E), followed by extension to the symmetric partner (PCeV or PCeV′) (F). Once connected, the DLV branches once more (G, small arrowhead) to form the trans-choroid plexus branch (TCB) (small arrowhead). By 120 hpf, the main vasculature of the mCP is in place (H). A diagrammatic representation of the final structure, with naming of the vessels and a superimposed mCP for comparison is shown (I). Small connecting vessels (concave arrow) connecting the DLV and TCB elaboration to form the mCP (J). Abbreviations: mesencephalic vein (MsV & MsV′), middle cerebral vessel (MCeV and MCeV′), dorsal longitudinal vein (DLV), posterior cerebral vein (PCeV and PCeV′) and myelencephalic choroid plexus (mCP). All images are oriented anterior to the left, and scale bars are 50 µm.

We further characterized mCP vasculature by describing the development of the DLV in more detail utilizing the Tg(*fli1*:eGFP) zebrafish transgenic line that labels vascular endothelial cells with GFP [12]. The DLV begins development between 40–48 hpf by sprouting from the Medial Cerebral Vein (MCeV and MCeV′)(Fig. 4B). The DLV extends by angiogenesis extending a growth cone like structure (Fig. 4C), until it reaches a hypothesized decision point (Fig. 4D). At this time, blood can be seen within the lumenized vessel, but remains static as it lacks a functional vascular outlet (data not shown). The vessels of the PCeV and PCeV′ migrate dorsomedially to meet the DLV (Fig. 4D), appearing to be attracted to the locale independent of the DLV (Fig. S1E). The DLV then develops preferentially to one of the PceV's where this vessel will connect and establish blood flow (Fig. 4E). A branch will then proceed toward the other PCeV and connect (Fig. 4F). The connection of the DLV and PCeV's approximately corresponds to the initial point at which GFP-expressing cells are observed in the Et^Mn16^ line. At around the same time, the MCeV-DLV junction fuses with the Mesencephalic Vein (MsV) forming the Dorsal Midline Junction (DMJ) (Fig. 4E–G). Concurrently, both the MsV and the MCeV supply the DLV with blood, with the MsV becoming the primary supply by 120 hpf (data not shown). Lastly, a cross-vein will form between the DLV-PCeV junction and the DLV-PCeV′ junction crossing the mCP between 3 and 5 dpf (Fig. 4G,H). We label this previously unannotated cross-vein, the trans-choroid plexus branch (TCB) as it transits the mCP. Further elaboration of the small vessels that supply the mCP continues past 5 dpf. This network is highly variable with vessels extending between the DLV and TCB (Fig. 4H and Fig. S1).

### The Notch signaling pathway in mCP development

**Figure 5:**
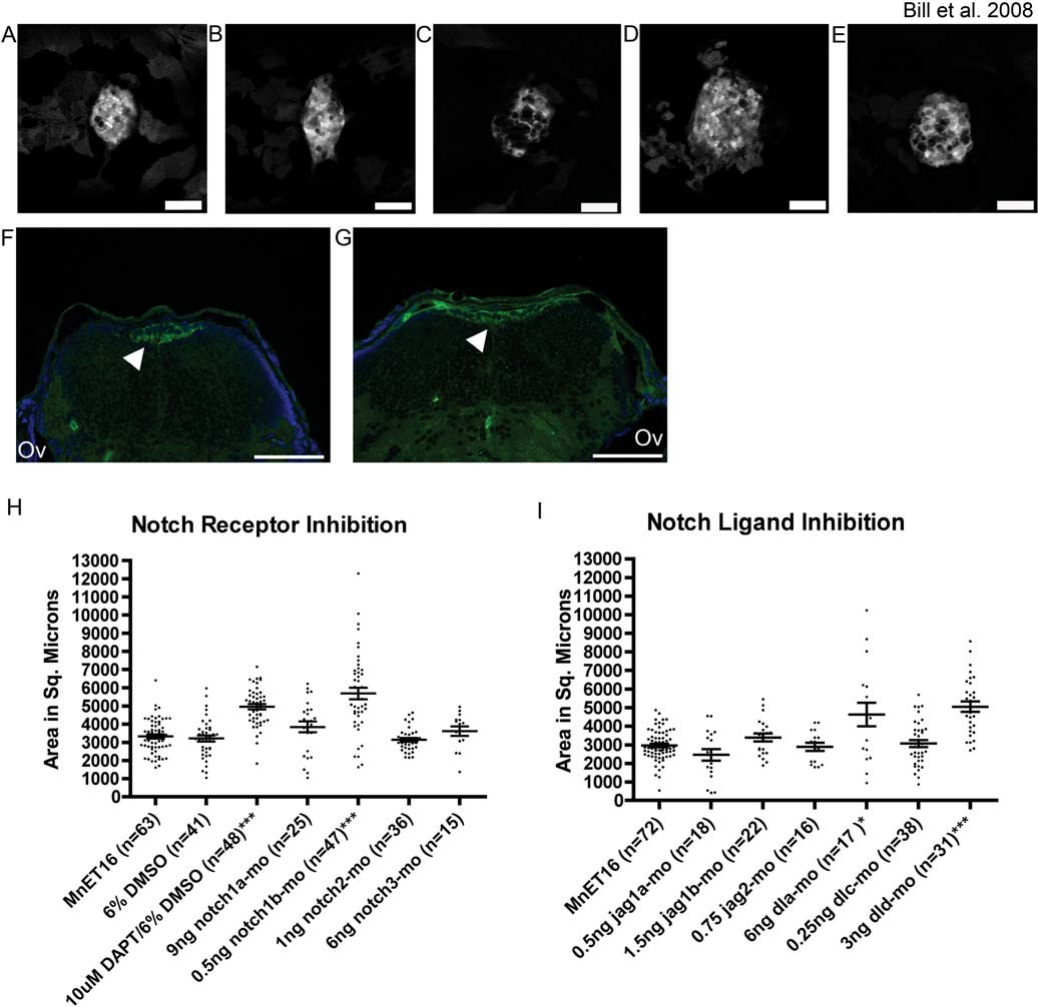
Notch signaling is required for proper development of the myelencephalic choroid plexus. Pan Notch Inhibition with DAPT (B, dorsal-mounted live larvae and G, transverse section) results in an increase in the mCP epithelial domain compared to vehicle-treated control larvae (A, dorsal mounted live larvae and F, transverse section). This increase in domain size is due to lateral spreading as the mCP remains as a monolayer (G, DAPT-treated versus F, vehicle treated). Further analysis showed that this effect is mediated by inhibition of *notch1b* (D, 5 dpf live larvae) *dla,* and *dld* (E, 5 dpf live larvae). Inhibition of *notch1a* (C, 5 dpf live larvae) did not significantly alter the size of the mCP epithelial domain but did effect overt structure. Panels A–F are dorsal views with anterior to the left, and panels G and H are 6 µm cryosections labeled with an antibody against GFP (green) and DAPI (blue) staining the nuclei. Quantitative measurements show the distribution mCP sizes in individual fish. Measurements are shown for Notch receptor inhibition by DAPT and morpholino experiments (H), and for Notch ligand inhibition by morpholinos (I) Each point in the histograms represents a measurement of a live larval zebrafish mCP. The mean±s.e.m. is indicated by the line and error bars respectively. Significant effects on mCP size are observed for 10 µM DAPT, *notch1b*, *dla*, and *dld* knockdown (* p = 0.02 and *** p<0.0001). For a full list of mean, s.e.m., and p-values, see table S2. Abbreviations: eye (E), pectoral fin (Pf), and otic vesicle (Ov). Arrows indicate mCP. Scale bars are 50 µm.

Multiple Notch ligands are expressed within the developing mammalian CP [13], with no known requirement for Notch signaling yet described. We used the gamma secretase inhibitor N-[N-(3,5-Difluorophenacetyl-L-alanyl)]-S-phenylglycine *t*-butyl ester (DAPT) [14] for a temporal-specific knockdown of Notch signaling during CP formation. DAPT was applied to embryos at 50 hpf to avoid indirect effects caused by early embryologic patterning defects. DAPT treatment resulted in a dramatic increase in mCP size in Et^Mn16^ larvae [from a mean of 3200 µm±160 s.e.m. in vehicle-treated Et^Mn16^ to a mean size of 5000 µm±150 s.e.m. (p = 1.98*10^−11^) in DAPT-treated larvae (Fig. 5H)]. This appears to be due to a lateral expansion as the mCP remains a monolayer (Fig. 5F).

As DAPT is a pan-Notch signaling inhibitor, we sought to specifically identify the individual Notch receptor and its ligand(s) involved in mCP formation. We conducted a reverse genetic screen utilizing morpholino oligonucleotides previously designed and validated against ten known zebrafish Notch pathway members [15]–[19]. We first screened morpholinos targeting four Notch receptors: *notch1a*, *notch1b*, *notch2*, and *notch3* (Fig. 5H). Treatment with 0.5 ng of *notch1b*-targeted morpholino leads to expansion of the mCP epithelia (mean 5700 µm±320 s.e.m. p = 3.8*10^−9^) (Fig. 5D) compared to wild type (3300 µm±120 s.e.m.)(Fig. 5A). We then screened morpholinos targeting Notch ligands: *jagged1a*, *jagged1b*, *jagged2*, *deltaA* (*dla*), and *deltaD* (*dld*) (Fig. 5I). The mCP in *dld*-targeted morphant larvae is significantly larger (5100 µm±280 s.e.m) compared to untreated Et^Mn16^ controls (3000 µm±95 s.e.m.)(p = 1.9*10^−8^) (Fig. 5E). Knockdown of *dla* also increases the mCP size significantly (4600 µm±630 s.e.m. p = 0.02), but this effect is much less consistent than the effect of *dld* (Fig. 5I). In addition, while the size of the mCP epithelial domain is not significantly different in *notch1a*-targeted morphants compared to controls, there is a visible effect on the mCP structure in morphant larvae (Fig. 5C) with a loss of a cohesive mCP epithelial sheet. Therefore, we conclude that knockdown of *notch1b*, *dla,* and *dld* significantly expands the domain occupied by the mCP epithelia. Together, these analyses implicate a novel Notch pathway critical role in CP formation.

## Discussion

### Identification of the CP as visualized in the Et^Mn16^ larvae

We demonstrate that the GFP-expressing cells of Et^Mn16^ are dCP and mCP epithelium. The mCP in zebrafish is located within the fourth ventricle posterior to the cerebellum (Fig. 1E,F), consistent with the location of the 4vCP/mCP in mammals and other teleosts [1], [20]. The mCP of zebrafish expresses Gfap, a transient fetal marker for human 4vCP [7]. This is the first description of the CP in zebrafish.

### Development of the mCP epithelia

We describe Et^Mn16^ GFP-expressing cells that migrate laterally along the roof plate of the fourth ventricle, coalesce and become a subset of the definitive mCP epithelia. Garcia-Lecea and coworkers describe a second enhancer trap SqET33-E20 (Gateways) transgenic zebrafish that express GFP within cells that migrate along the anterior posterior axis at the midline, coalesce and form a distinct subset of definitive mCP epithelia [21]. These SqET33-E20-labeled cells are temporally and spatially separated from the cells we describe marked by Et^Mn16^. The Et^Mn16^ transgenic line labels cells highly reminiscent of cell lineages described by Hunter and Dymecki that migrate out of the dorsal rhombomeric lip and directly populate the mouse mCP [22]. We conclude that these migrating cells found in fish and mice are orthologous and the resulting developmental model represents a likely shared and conserved mechanism for CP development in vertebrates.

### The vasculature of the mCP is supplied by the DLV

We characterize the mCP-associated vasculature in zebrafish. The DLV supplies the blood to the mCP, bifurcating just inside the margin of the mCP epithelia, and forming junctions with both the PCeV and PCeV′ (Fig. 3A–F). The DLV bends dorsally just before crossing the mCP margin and is tightly associated with the dorsal surface of the mCP epithelial monolayer (Fig. 3G,H). The DLV sprouts from the MCeV and MCeV′ between 40–48 hpf via angiogenesis. The DLV reaches a hypothesized decision point at ∼50 hpf, and the PCeV and PCeV′ grow dorsomedially to meet it. The trans-choroid plexus branch (TCB) develops between the DLV-PCeV junction and the DLV-PCeV′ junction between 55–120 hpf. This forms a variable structure that overlays the mCP epithelia, and produces a network of small vessels that connect the bifurcated DLV and TCB. This is the first description of the development of the vasculature associated with the mCP and has many similarities to the development of mammalian mCP development [9]. Future studies will be required to identify whether these small vessels that cross between the DLV and TCB are orthologous to the fenestrated capillaries of mammals.

### Synthesis of a model for zebrafish mCP development

By comparing the developmental time points at which the DLV and mCP epithelia develop (Fig. 2, Fig. 4) [21], we can precisely order developmental events in the mCP. Garcia-Lecea and coworkers describe a subset of mCP epithelial precursor cells that are initially spread out at the dorsal midline from rhombomere 2–6 that coalesce between 48–72 hpf and express GFP in their SqET33-E20 line [21]. The DLV and PCeV develop to the location associated with the mCP synchronously with the GFP-expressing cells of SqET33-E20. Intriguingly, the developmental cue is independent of the vasculature (Fig. S1E) suggesting an attractant that promotes growth to the fourth ventricle roof plate. Previous work has suggested the existence of such an organizer in mouse [23]–[26]. After the DLV has completed junction formation with the PCeV and PCeV′, the subset of Et^Mn16^ defined mCP migrate laterally coalescing on the roof of the fourth ventricle, by 5 dpf. This model is the first to integrate both the cellular migration of the mCP epithelial precursors with that of the development of the vasculature and lays the groundwork for further vascular studies. For example, the zebrafish should be a very tractable model to identify, the dorsal roof organizer and the factors that localize both vasculature and epithelial development to the fourth ventricle roof plate.

### The Notch signaling pathway is required for mCP development

We undertook a small-scale targeted chemical and morpholino-mediated screen to investigate the role of Notch signaling in mCP development. The pan-Notch signaling chemical inhibitor (DAPT), when exposed to 50 hpf Et^Mn16^ embryos increased the size of the developing mCP epithelia. To specify those members of the Notch pathway that are involved, we utilized previously validated Notch and Notch ligand targeting morpholinos to investigate the individual effect of several notch receptors and ligands. The *notch1b, dla, and dld*-targeted mopholinos increase the size of the mCP similar to the DAPT treatment, while other Notch and Notch ligand-targeting morpholinos did not significantly alter mCP size. It should be noted, however, that while other morpholinos were unable to change CP size, there may have been other effects on the structure as exemplified by a *notch1a* knockdown mCP (Fig. 5c) that has an increase in GFP-negative surface area within the mCP structure. Previous reports on Notch signaling in the mammalian CP have shown that multiple Notch ligands are expressed in the CP [13], a novel Notch2-mediated ligand independent role for adult rat CP integrity [27], and that expression of a constitutively activated Notch1 protein causes an increase in the proliferative potential of the CP precursor cells by activating mitosis in normally quiescent cells [22]. This study presented here identifies an endogenous requirement for *notch1b* in development of the mCP and a functional role for any Notch ligand (*dla* and *dld*) in the mCP. Future studies will be required to determine the relationship of Notch1a, Dla, and Dld, the localization of these proteins, and the nature of the expanded mCP including the role of the vasculature in determining the domain occupied by the CP.

### Conclusion

We provide the first description of the zebrafish CP by characterizing a transgenic line (Et^Mn16^) that expresses GFP in the zebrafish dCP and mCP epithelia. We observe a tight integration between the mCP and its vascular supply, primarily the DLV and TCB. We provide a model that integrates development of the epithelial and vascular components of the mCP. In addition, we demonstrate a functional role for endogenous Notch signaling in mCP epithelia. We identify a Notch receptor and two Notch ligands involved in the lateral expansion of the mCP epithelial layer. This model provides a unique system in which to rapidly expand the knowledge of the molecular signaling pathways that contribute to CP development.

## Methods

### Fish Strain, Care and Use

Fish are housed in the University of Minnesota Zebrafish Core facility under standard conditions [28] in accordance with IACUC-approved protocols. Lines propagated for this study include: Tg(*fli-1*:eGFP)[12] (ZIRC), Tg(*gata-1*:dsRed)[10] (a kind gift from Dr. Len Zon), and Et^Mn16^ (see below). Double transgenics were developed by paired matings and include: Tg(*fli-1*:eGFP)/Tg(*gata-1*:dsRed) and Tg(*gata-1*:dsRed/Et^Mn16^). Wild type zebrafish are obtained from Segrest Farms. Timed bulk matings were used to obtain one-cell embryos for injections.

### Creation of the Et^Mn16^ line

Et^Mn16^ is a product of a Sleeping Beauty (SB) transposon mediated-enhancer trap screen previously described [6]. In brief, a weak EF1α enhancer element upstream of zebrafish optimized GFP (GM2) was placed in between the SB inverted-direct repeats (pT2/S2EF1a-GM2). Co-injection of this construct with SB transposase mRNA was performed into 1–2 cell embryos and propagated as described [29], [30].

### Immunohistochemisty

Six micron cryosections were obtained on a Leica CM3050S cryostat via previously developed techniques [31]. Antibodies used include zrf-1[8], [32] (1∶5, ZIRC), anti-GFP G1544 (1∶200, Sigma), and Alexafluor conjugated goat anti-mouse and goat anti-rabbit (1∶2000, Invitrogen). Slides were mounted with Vectashield Mounting Medium with DAPI (Vector Laboratories).

### Microinjection of morpholinos

Zebrafish were collected and injected as described previously [33]. Morpholino oligonucleotides were obtained from GeneTools LLC. To investigate Notch signaling during CP formation, we used published oligonucleotides against Notch, Jagged and Delta transcripts from the zebrafish (Table S1) [15]–[19]. For each morpholino, we confirmed that there was no significant sequence overlap between oligonucleotides targeting these highly related proteins (Table S1). The *notch1b*-targeted morpholino was co-injected with *tp53*-targeted morpholino (1.5×) to verify effects were not due to off-targeting [34].

### Notch inhibitor treatment

We used the gamma-secretase inhibitor DAPT (Sigma) to inhibit all Notch signaling in zebrafish [14]. To identify the window of Notch sensitivity, we added DAPT at several different developmental time-points (data not shown). We determined treatment at 50 hours post fertilization was optimal. Fish were dechorinated and placed in medium Petri dishes with 6ml of embryo medium. 100 µM DAPT (suspended in DMSO) was added at various doses as described previously [35]. 6% DMSO in embryo medium (v/v) was used as a vehicle control.

### Microscopy and Imaging

All images were taken on a Zeiss Axioplan 2 with Apotome utilizing the Axiocam mRM (Zeiss). For brightfield microscopy, fish were imaged under DIC optics. For imaging CP development, zebrafish were mounted in 1% low melting point and anesthetized using tricane [28]. Images were processed using Axiovision software with Image 4D (Versions 4.2 and 4.6; Zeiss), and Photoshop (Version 8; Adobe).

### Measurements and Statistics

The surface area of the mCP (as defined by Et^Mn16^ fluorescence) was measured using the outline measurement module (Axiovision 4.2 and 4.6). Values were entered into graphing software, Prism (Version 4; GraphPad) for histograms and normality statistics. Normality was determined for each of the distributions utilizing the D'Agostino & Pearson omnibus normality test (alpha = 0.05) and Shapiro-Wilk normality test (alpha = 0.05) (data not shown) for each of the treatments to verify that the Student T-test was appropriate (Version 4; GraphPad). Student T-tests were performed to determine statistical significance (two tailed with unequal distribution) in Excel (version 11.4.1; Microsoft).

## Supporting Information

Figure S1 Variation of the DLV-PCeV junction. The DLV (arrows) bifurcated to join the PCeV and PCeV′ (open arrowheads) and the TCB (small arrowheads) connects the two junctions. This structure is plastic. The most common structure includes a third branch that transits between the DLV and the TCB (A–C), but in some instances the third DLV-TCB branch does not form by 5 dpf (D). The most rare occurrence is that the DLV does not develop (E). The PCeV and PCeV′ still meet in the location of the pCP, but lack blood flow (E, open arrowheads). All images are shown with anterior to the left. Scale bar is 50 µm.
(1.03 MB TIF)

Table S1 (0.05 MB RTF)

Table S2 (0.05 MB DOC)
