# Supplementary figures and images for: Development and Notch Signaling Requirements of the Zebrafish Choroid Plexus
Source: PLoS One. 2008 Sep 3;3(9):e3114. doi: 10.1371/journal.pone.0003114 (PMC2528000; doi:10.1371/journal.pone.0003114)

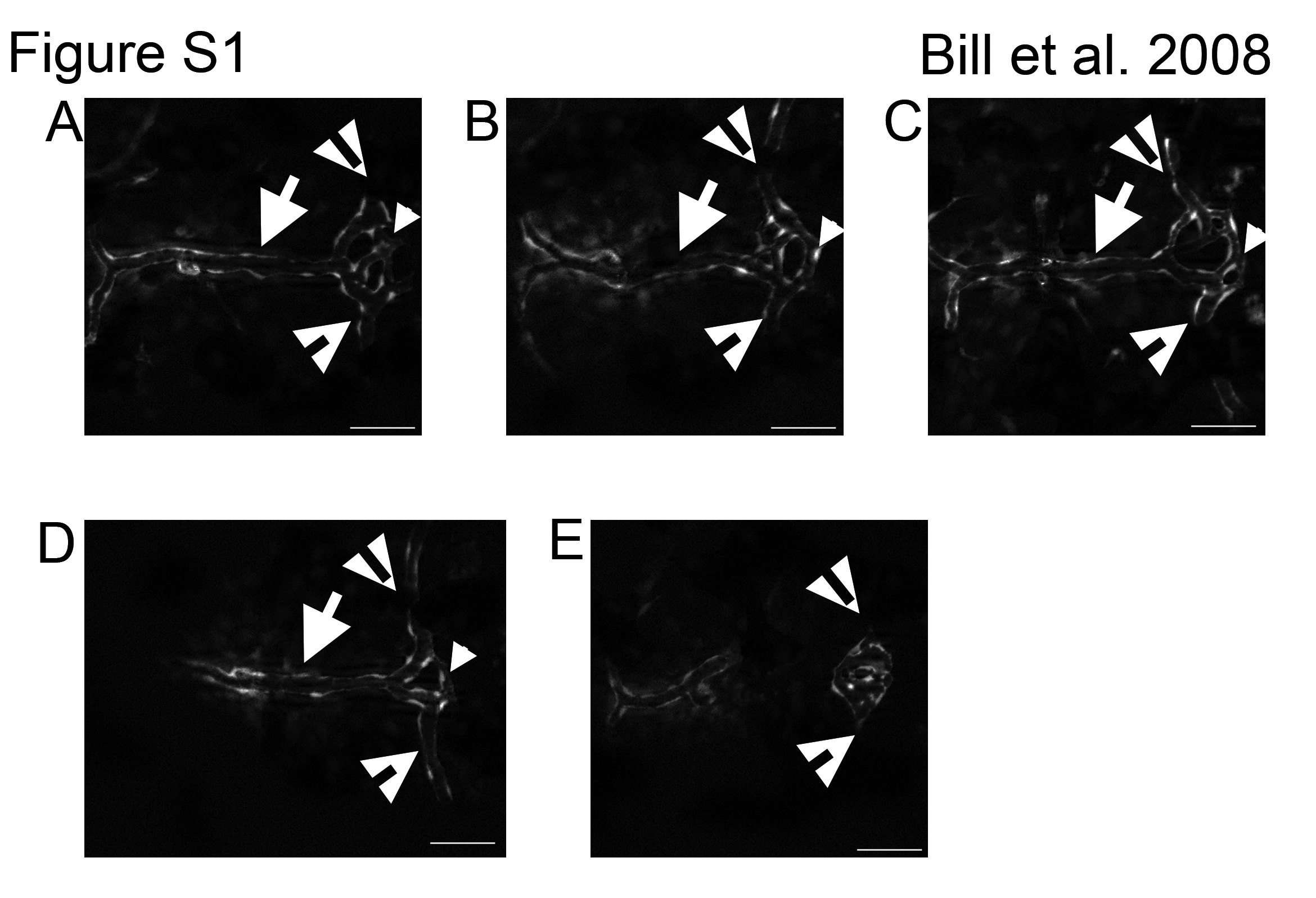

Supplement: Figure S1 — Variation of the DLV-PCeV junction. The DLV (arrows) bifurcated to join the PCeV and PCeV′ (open arrowheads) and the TCB (small arrowheads) connects the two junctions. This structure is plastic. The most common structure includes a third branch that transits between the DLV and the TCB (A–C), but in some instances the third DLV-TCB branch does not form by 5 dpf (D). The most rare occurrence is that the DLV does not develop (E). The PCeV and PCeV′ still meet in the location of the pCP, but lack blood flow (E, open arrowheads). All images are shown with anterior to the left. Scale bar is 50 µm. (1.03 MB TIF) [file pone.0003114.s001.tif]
