# Supplementary material for: Development and Notch Signaling Requirements of the Zebrafish Choroid Plexus
Source: PLoS One. 2008 Sep 3;3(9):e3114. doi: 10.1371/journal.pone.0003114 (PMC2528000; doi:10.1371/journal.pone.0003114)
Supplement: Table S1 — (0.05 MB RTF) [file pone.0003114.s002.doc]

**Table S1**: Morpholinos used in this study.

| Target Gene | Morpholino sequence | Maximun Match score | Match description for Scores greater than 40. | Citation |
| --- | --- | --- | --- | --- |
| notch1a | GAAACGGTTCATAACTCCGCTCGG | jagged2, DeltaD (50) | 12bp noncontiguous |  |
| notch1b | ATACGTATAGTGGACTAGGAGAAAGA | notch2, deltaC & D, (24) | na |  |
| notch2 | AGGTGAACACTTACTTCATGCCAAA | DeltaD(32) | na |  |
| notch3 | ATATCCAAAGGCTGTAATTCCCCAT | Jagged2 (36) | na |  |
| jagged1a | CGGTTTGTCTGTCTGTGTGTCTGTC | DeltaA (32) | na |  |
| jagged1b | CTGAACTCCGTCGCAGAATCATGCC | DeltaD (44) | 11bp noncontiguous |  |
| jagged2 | TCCTGATACAATTCCACATGCCGCC | Notch1a (50) | 12bp noncontiguous |  |
| deltaA | CTTCTCTTTTCGCCGACTGATTCAT | Jagged1a (32) | na |  |
| deltaC | AGCCATCTTTGCCTTCTTGTCTGCT | DeltaD (28) | na |  |
| deltaD | GGTTTTGGACTTACCTCGGTTGCAA | Notch1a (50) | 12bp noncontiguous |  |
