# Supplementary material for: Development and Notch Signaling Requirements of the Zebrafish Choroid Plexus
Source: PLoS One. 2008 Sep 3;3(9):e3114. doi: 10.1371/journal.pone.0003114 (PMC2528000; doi:10.1371/journal.pone.0003114)
Supplement: Table S2 — (0.05 MB DOC) [file pone.0003114.s003.doc]

**Table S2: Statistics from Notch Inhibition Assays.**

For Notch Receptor inhibitors (Figure 5h)

| Treatment | Mean (μm) | s.e.m. | p value, student t-test comparison to MnET16 unless noted. |
| --- | --- | --- | --- |
| MnET16 | 3300 | 120 | na |
| 6% DMSO (Vehicle) | 3200 | 160 | 0.6 |
| 10μM DAPT | 5000 | 150 | 2*10^-11^ (Compared to vehicle) |
| 9ng *notch1a*-mo | 3800 | 300 | 0.11 |
| 0.5ng *notch1b*-mo | 5700 | 320 | 3.8*10^-9^ |
| 1ng *notch2*-mo | 3100 | 100 | 0.26 |
| 6ng *notch3*-mo | 3600 | 260 | 0.31 |

For Notch Ligand inhibitors (Figure 5i)

| Treatment | Mean (μm) | s.e.m. | p value, student t-test comparison to MnET16. |
| --- | --- | --- | --- |
| MnET16 | 3000 | 95 | na |
| 0.5 *Jag1a*-mo | 2500 | 310 | 0.14 |
| 1.5ng *Jag1b*-mo | 3400 | 210 | 0.07 |
| 0.75 *Jag2*-mo | 2900 | 220 | 0.78 |
| 6ng *dla*-mo | 4600 | 630 | 0.02 |
| 0.25ng *dlc*-mo | 3100 | 190 | 0.72 |
| 3ng *dld*-mo | 5100 | 280 | 1.9*10^-8^ |

na = not applicable
